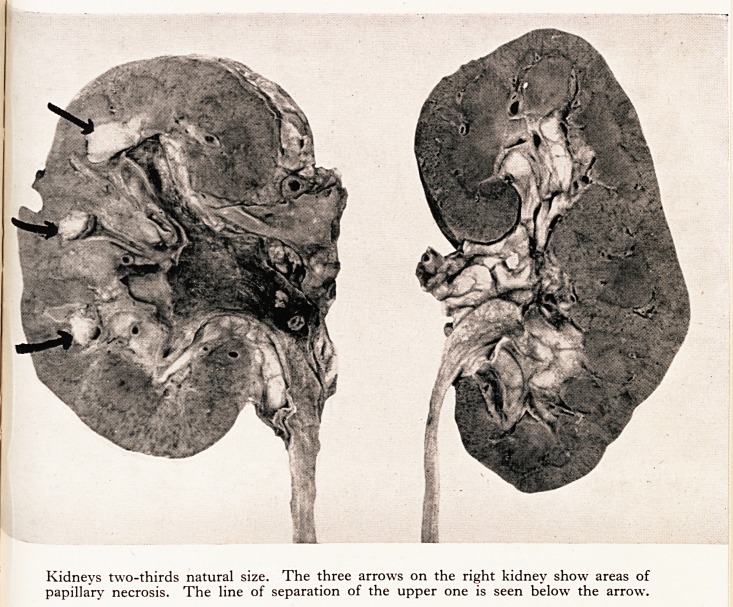# Unilateral Papillary Necrosis of Kidney in a Diabetic
*A case report at a Clinico-Pathological Conference held on 26th February, 1952, in the Department of Pathology, University of Bristol.


**Published:** 1952-07

**Authors:** 


### UNILATERAL PAPILLARY NECROSIS OF KIDNEY IN A DIABETIC*

### A rare complication of enlargement of the prostate gland

**Dr. B. E. McConnell**—This old man was an N.H.S. patient of mine. He was the proprietor of four or five taxi-cabs, and still drove himself at the age of 75. He led an extremely active life up till admission to hospital.

I saw him first, eighteen months ago when he had epididymo-orchitis, for which I gave sulphamezathine. He refused to go to hospital because of driving his taxi. I did not test his urine because I saw him at home and not at my consulting rooms. In July 1951 he had dysuria and a recurrence of epididymo-orchitis; he agreed to go to the Genito-urinary out-patients, where glycosuria was discovered and he was advised by Mr. Ashton Miller to enter hospital for trans-urethral resection of his enlarged prostate.

His diabetes was controlled quite easily by diet. He was under investigation as an out-patient until he was admitted as an emergency on October 9th, 1951, because he suddenly developed severe abdominal pain. He was only very slightly febrile. He was definitely tender in the right iliac fossa. I felt that the diagnosis lay between appendicitis and renal infection.

**Mr. A. Wilfrid Adams**—I saw this man after his admission in October. There was right-sided tenderness and " belly-ache ". I suspected kidney trouble. There was slight haematuria and the kidney region was tender, but there was no clear indication of the aetiology. Much sugar was found in his urine and he was given insulin. An intravenous pyelogram showed no function of the right kidney which was surprising because a previous pyelogram performed when he was seen in the out-patient department in July 1951 had shown both kidneys working normally. The blood pressure was 150/90 and the blood sugar 600 mgm. On cystoscopy an oedematous right ureteric meatus was seen. No urine came from it. A swelling in the intramural portion was opened and a lot of debris, followed by clear urine, escaped This urine contained pus cells and *B. coli.* The next day he was better but had a rigor after a period of right-sided back-ache. On October 24th, the blood urea was 32 mgm%. He was given penicillin and streptomycin. The tenderness in the loin was now nearly gone. On November 6th I explored the right kidney surgically because of clinical evidence of tension. Examination of the kidney was difficult because of dense perinephric fibrosis and as the pelvis was only slightly distended I drained the kidney by a stab wound through the kidney substance into the pelvis. I did this nephrostomy in the hope that free drainage would allow the kidney to function again.

He became uraemic (blood urea up to 200 mgm%) and deteriorated. I was rather mystified as to what was happening. Pain in the right lower abdomen persisted and the house surgeon, Mr Zabron, felt a hard swelling in the iliac fossa which, he declared, was pulsating. I was not very much impressed by it. On November 15th the patient died.

**Dr. J. E. Cates**—I have very little to add from the medical point of view. There were two sides to his case. Firstly he was a diabetic who was sick, and diabetics who are sick or have operations we like to control on insulin four times a day with fixed amounts of carbohydrates in their meals. His diabetes was controlled in this way and gave no anxiety.

Secondly, when a diabetic gets uraemia there are two things to think of; one is what is now called Kimmelstiel-Wilson kidney, a condition associated with high blood pressure, protein in the urine and peripheral vascular disease. The second is much rarer, but we can talk about it after Professor Hewer tells us his findings at post-mortem.

**Professor T. F. Hewer** reported the autopsy—I found a fresh operation wound in the right iliac fossa, from which blood-stained fluid escaped. There was oedema of the lungs, slight enlargement of the heart and vascular evidence of hypertension. The chief interest was in the kidneys.

* A case report at a Clinico-Pathological Conference held on 26th February, 1952, in the Department of Pathology, University of Bristol.

PLATE IV

Kidneys two-thirds natural size. The three arrows on the right kidney show areas of papillary necrosis. The line of separation of the upper one is seen below the arrow.

The left kidney looked healthy, except for some fine scarring of the cortex and microscopic hypertensive vascular changes. It was free from infection. There was no glomerular sclerosis of the Kimmelstiel-Wilson type.

The right kidney I cut just in front of the surgical incision (*see* Plate IV). The drainage tube lay in the pelvis which was acutely inflamed. There was evident pyelonephritis, with abscesses on the surface and scarring. The remarkable thing was necrosis of the tips of the papillae. These were well demarcated and absolutely necrotic: they are indicated by arrows in the photograph and can be seen to be undergoing separation, as sloughs, from the adjacent medulla.

The right ureter was dilated, because where it ran across the common iliac artery it was involved in some fibrous tissue around an atheromatous aneurysm of this vessel. There was extensive atheroma of the abdominal aorta with the usual calcification, and no suggestion of syphilitic aortitis. Below the point where the ureter was obstructed there was no dilatation and the ureteric orifice was patent. The bladder wall was hypertrophied and there was an acute purulent cystitis. The prostate was enlarged and there was an old fibrous epididymo-orchitis on the left side only. The unilateral ureteric obstruction undoubtedly accounted for the unilateral pyelonephritis.

Histologically these pale necrotic papillae looked like infarcts, with a perfectly clear line of demarcation from the viable medulla in which there were some lymphocytes and plasma cells but few polymorphonuclears. Apart from this the right kidney showed only an acute pyelonephritis and hypertensive arteriolar changes. There were no evident vascular lesions to explain the papillary necrosis.

*Papillitis renis necroticans*, as this condition is called, was well reviewed by Edmondson *et al.* (1947) who studied 32,000 routine autopsies, of which 859 were diabetic. The incidence of acute pyelonephritis in the non-diabetics was 3·28 per cent and in the diabetics 12·45 per cent. The incidence of this curious papillary necrosis in the non-diabetics with pyelonephritis was 2 per cent, and in all but one of these there was chronic urinary obstruction. In the diabetics with pyelonephritis papillary necrosis occurred in 27 per cent so there is a significantly greater incidence in diabetics. It is probable that this is due to the associated renal vascular lesions in diabetics.

There is no very satisfactory explanation of the mechanism of production of this curiously localized necrosis: it does not conform with any arterial distribution and the very slight local inflammatory reaction cannot explain it. I wonder whether it may be venous in origin and in some way related to the phenomenon of pyelo-venous back-flow that is sometimes seen in ascending pyelography. I do not mean that this investigation induces it but that infection of the kidney, with increased pelvic pressure due to ureteric obstruction may interfere with venous drainage around the papillae and produce venous infarction. Because of their vascular disease and susceptibility to infection diabetics are particularly likely to suffer this accident.

In the present case some of the necrotic papillary tissue separated and was passed down the ureter, causing blockage at the lower end: that is my interpretation of the cystoscopy finding.

Death was due finally to a combination of uraemia, infection and pulmonary oedema. I might add that papillary necrosis is the second condition that Dr. Cates had in mind!

**Dr. J. E. Cates**—I am wondering why the atheromatous aneurysm should have fibrous tissue round it.

**Professor T. F. Hewer**—I think it had been leaking into the tissues round about. There was a discoloured mass with a lot of thrombus. I have seen fibrosis around leaking atheromatous aneurysms.

### REFERENCE

Edmondson (1947). *Arch. Int. Med.* **79**, 148.

## Figures and Tables

**Figure f1:**